# Retroperitoneal iliac conduits as an alternative access site for endovascular aortic repair: a tertiary care center experience

**DOI:** 10.1590/1677-5449.210033

**Published:** 2021-09-10

**Authors:** Rajesh Vijayvergiya, Lipi Uppal, Ganesh Kasinadhuni, Prafull Sharma, Ashish Sharma, Ajay Savlania, Anupam Lal

**Affiliations:** 1 Post Graduate Institute of Medical Education & Research – PGIMER, Chandigarh, India.

**Keywords:** aortic aneurysm, aortic dissection, endovascular aortic repair, iliac conduit, vascular access, aneurisma aórtico, dissecção aórtica, reparo endovascular de aneurisma, conduto ilíaco, acesso vascular

## Abstract

**Background:**

Retroperitoneal open iliac conduits (ROIC) are used in patients with hostile iliac anatomy undergoing endovascular aortic repair (EVAR).

**Objectives:**

We hereby report our experience of ROIC in patients subjected to EVAR.

**Methods:**

This was a retrospective evaluation of 8 patients out of a total of 75 patients (11%) who underwent EVAR in the last 10 years. Pre-procedure computed tomography angiography was used to assess the dimensions of iliac and femoral arteries. Patients who had small arterial dimensions (i.e. smaller than the recommended access size for the aortic endograft device) were subjected to ROIC.

**Results:**

The mean age of the 3 males and 5 females studied was 45.7 ± 15.2 years. The indication for ROIC was the small caliber ilio-femoral access site in 7 patients and atherosclerotic disease in 1 patient. All external grafts were anastomosed to the right common iliac artery except one which was anastomosed to the aortic bifurcation site because of a small common iliac artery. The procedural success rate was 100%. Local access site complications included infection (n=1), retroperitoneal hematoma (n=1), and need for blood transfusion (n=3). The median post-intervention hospital stay was 10 days. All patients had favorable long-term outcomes at a median follow-up of 18 months.

**Conclusions:**

Female patients require ROIC during EVAR more frequently. Adjunctive use of iliac conduit for EVAR was associated with favorable perioperative and short-term outcomes.

## Resumo

**Contexto:**

Os condutos ilíacos abertos retroperitoneais são utilizados em pacientes submetidos a reparo endovascular de aneurisma (REVA) com anatomia ilíaca hostil.

**Objetivos:**

Relatamos a nossa experiência com os condutos ilíacos em pacientes submetidos a REVA.

**Métodos:**

Trata-se de uma avaliação retrospectiva de oito pacientes, de um total de 75 (11%), os quais foram submetidos a REVA nos últimos 10 anos. Foi realizada angiotomografia computadorizada antes do procedimento para avaliar as dimensões das artérias ilíaca e femoral. Os pacientes com dimensões arteriais menores, abaixo do tamanho de acesso recomendado para o dispositivo de endoprótese aórtica, foram submetidos a condutos ilíacos.

**Resultados:**

A média de idade dos participantes foi de 45,7±15,2 anos, sendo três do sexo masculino e cinco do sexo feminino. As indicações para condutos ilíacos foram local de acesso ilíaco femoral de pequeno calibre, para sete pacientes, e doença aterosclerótica, para um paciente. Todas as próteses externas foram anastomosadas na artéria ilíaca comum direita, com exceção de uma, que foi anastomosada no local da bifurcação aórtica por apresentar artéria ilíaca comum menor. A taxa de sucesso do procedimento foi de 100%. As complicações no local de acesso incluíram infecção (n = 1), hematoma retroperitoneal (n = 1) e necessidade de transfusão de sangue (n = 3). O tempo mediano de internação hospitalar pós-intervenção foi de 10 dias. Todos os pacientes apresentaram desfechos de longo prazo favoráveis no seguimento mediano de 18 meses.

**Conclusões:**

As pacientes do sexo feminino necessitaram de condutos ilíacos durante REVA com maior frequência. O uso adjuvante de condutos ilíacos com REVA foi associado a desfechos perioperatórios e de curto prazo favoráveis.

## INTRODUCTION

Management of aortic diseases has significantly changed from open surgery to endovascular repair, over the last 2 decades. The endovascular approach is a minimally invasive intervention, having better perioperative morbidity and mortality, and similar 5-year results compared to open surgical repair.^1^ It is the mainstay of treatment for aortic aneurysms and Type-B aortic dissections.^1^^,^^2^ Endovascular aortic repair (EVAR) of aortic aneurysm and type-B aortic dissection utilizes the femoral access site for retrograde advancement of a stent-graft measuring from 18 - 24 F in dimension. Hostile iliac-femoral arterial anatomy hampers the maneuverability of these large-bore devices and consequently increases the risk of local vascular complications. To overcome such access site problems, open surgical or endovascular iliac conduits have been recommended.^3^ Retroperitoneal open iliac conduit (ROIC) is considered the most appropriate choice for unfavorable anatomy.^3^^,^^4^ We hereby report our experience of ROIC in patients with difficult vascular access subjected to EVAR.

## METHODS

This was a retrospective evaluation of 8 patients out of a total of 75 patients (11%), who underwent EVAR with ROIC at our institute in the last 10 years. The demographic profile, procedural indications, iliac vessel sizes, length of hospital stay, and short and long term outcomes were noted. Pre-procedure computed tomography (CT) angiography was used to assess the dimensions of iliac and femoral arteries. Patients who had small arterial dimensions (smaller than the recommended access size for the particular device) were subjected to ROIC. For the statistical analysis, continuous variables were summarized as mean ± 1 standard deviation (SD) or median interquartile range [IQR], based on the distribution. The study was in accordance with the Helsinki Convention and approved by the institutional ethics committee for the retrospective analysis, vide no. INT/IEC/2020/SPL-1400.

ROIC Surgical Procedure: The retroperitoneal space was entered through a short, oblique incision in the lower abdominal quadrant, above the inguinal ligament, performed under general anesthesia. Following the incision, division of the external oblique, internal oblique, and transversalis muscles was performed. The peritoneum along with its contents was retracted superiorly to expose the underlying common iliac artery (CIA), followed by external and internal iliac arteries. Following systemic anticoagulation, the vessels were clamped and an end-to-side conduit anastomosis was performed with either the CIA or distal aorta, as per the planned intervention. An 8 or 10 mm conduit was used. The distal end of the PTFE graft was thereafter clamped and access was obtained from the side of the prosthesis for introduction of the vascular sheath. After completion of the endovascular procedure, most of the conduit was excised, leaving a small stump near the anastomosis which was sutured with 6-0 Prolene suture to secure hemostasis. With this technique, a small amount of prosthetic material was however left *in situ*. Alternatively, the graft was used to facilitate ilio-femoral bypass in patients with severe peripheral arterial disease.

Post-intervention, a regular surgical wound dressing was performed and patients were discharged following satisfactory wound healing. A repeat CT angiography was performed in all patients within 6 months of intervention.

## RESULTS

**Figure 1:**
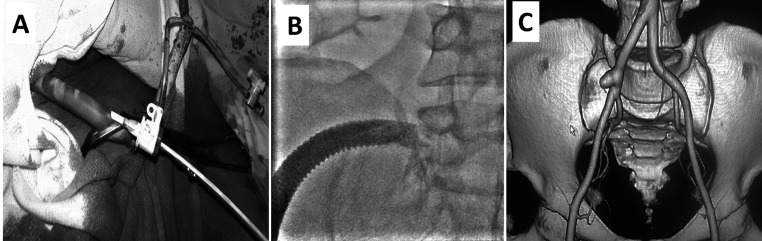
The Polytetrafluoroethylene (PTFE) graft **(A)** anastomosed with the right common iliac artery (CIA) **(B)** was visible. A follow-up 3-dimensional volume-rendered computed tomography (CT) angiographic image showed a small conduit stump attached to the right CIA **(C)**.

**Figure 2:**
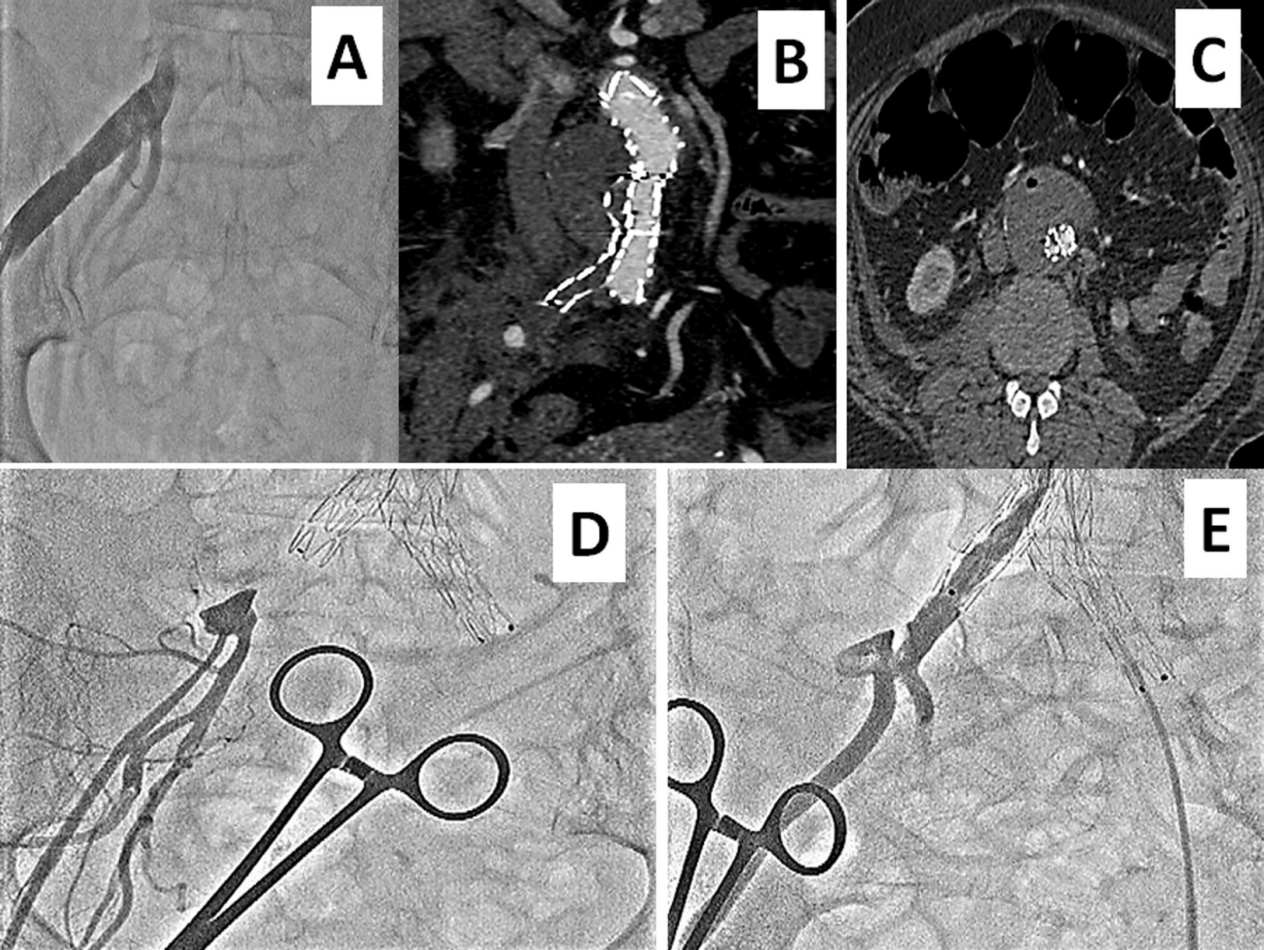
A PTFE conduit was anastomosed to the right CIA **(A)** in abdominal aortic aneurysm (AAA) case 4. Post-intervention, day-3 CT reconstructed coronal and axial images showed thrombotic occlusion of the right limb of the aortic stent graft **(B** & **C)**. Following balloon angioplasty and thrombus suction **(D)**, good flow was achieved across the occluded right limb **(E)**.

**Figure 3:**
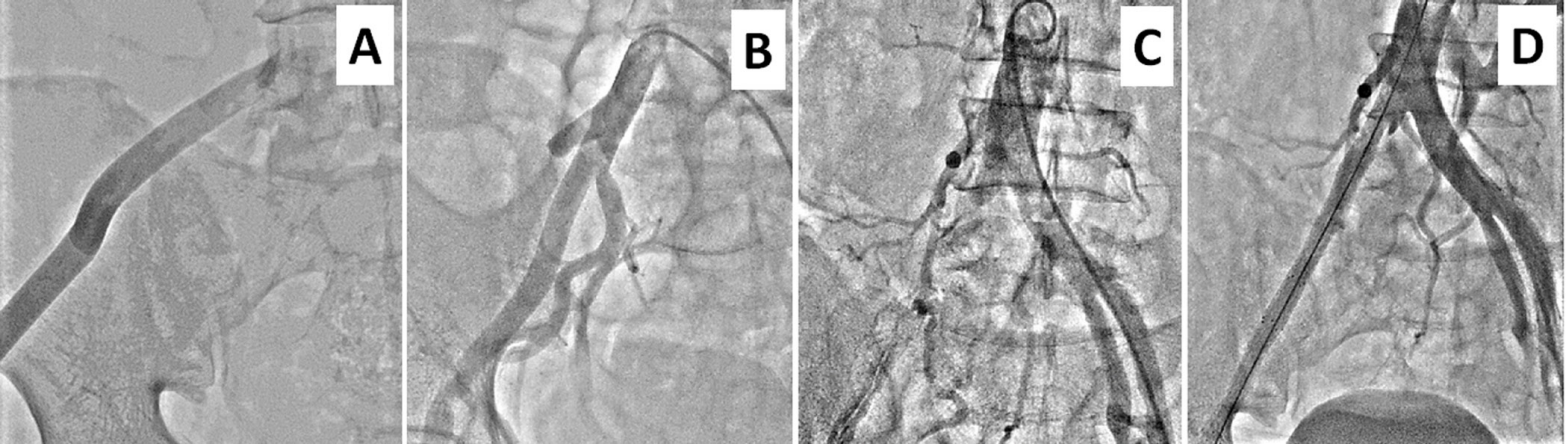
In thoracic aneurysm case 2, the conduit was anastomosed with the right CIA **(A)**, the residual stump of which could be seen attached to the CIA on a follow-up angiogram **(B)**. At 16 months of follow-up, the right CIA was atherosclerotic with total occlusion **(C)**, which was successfully stented **(D)**.

**Figure 4:**
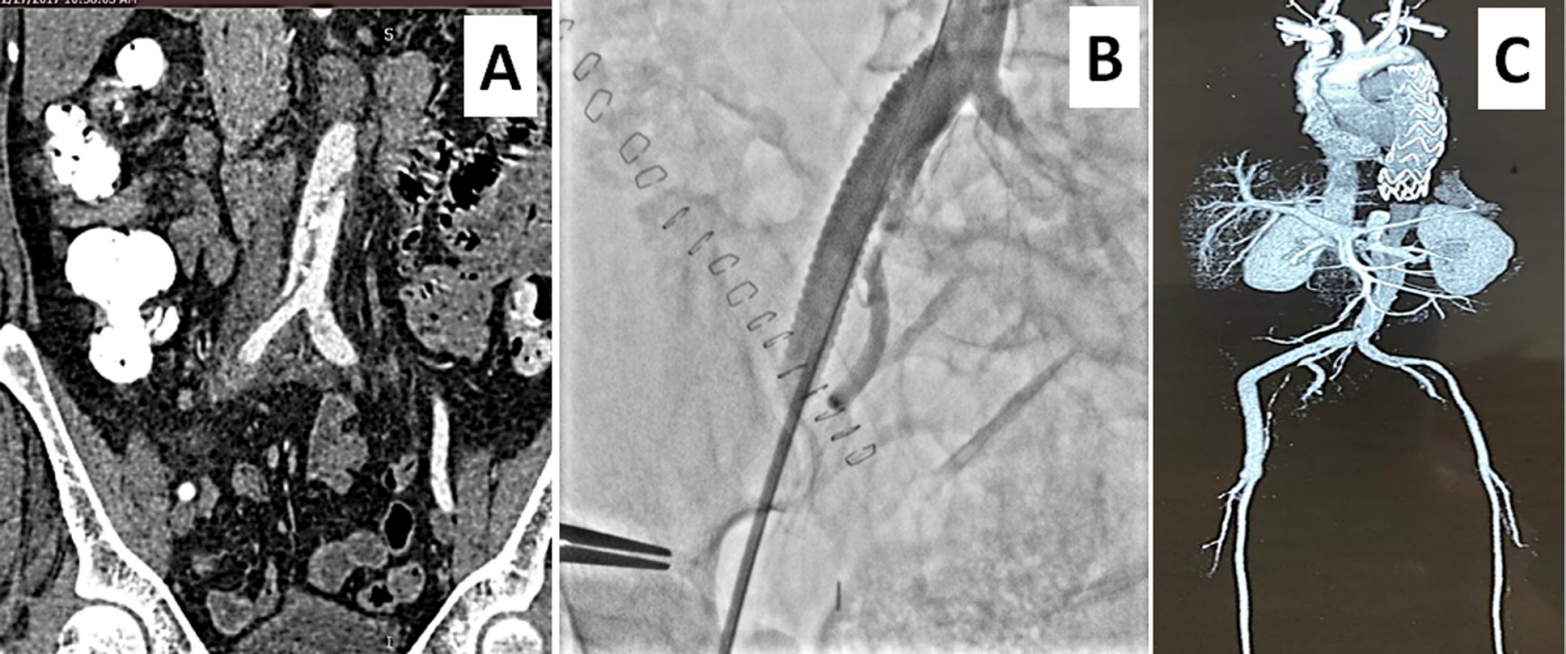
Coronal reconstructed CT image **(A)** of case 8 showed type-B aortic dissection extending up to left CIA and diseased right CIA. A contrast angiogram showed anastomosed conduit at the level of aortic bifurcation **(B).** A follow-up, 3-dimensional volume-rendered CT angiographic image showed patent aorto-right femoral bypass graft, diseased right CIA and patent descending thoracic aorta stent-graft **(C).**

The mean age of the 3 males and 5 females studied was 45.7 ± 15.2 years. Table 1 describes the 8 individual cases in detail. The descending thoracic aorta (DTA) was diseased in 6 cases and the infra-renal abdominal aorta in 2 cases. Hypertension and smoking were the two common risk factors. Two cases of thoracic pseudoaneurysm had underlying tuberculosis (case 5 and 7). The indication for ROIC was the small caliber ilio-femoral access site in 7 patients and atherosclerotic disease in 1 patient (case 8). The mean diameters of the right side CIA, external iliac artery (EIA), and common femoral artery (CFA) were 9.01 ± 2.11 mm, 6.22 ± 0.78 mm, and 6.27 ± 0.76 mm, respectively. All external grafts were anastomosed to the right CIA (Figure 1A, 1B, 2A, 3A) except one, which was anastomosed to the aorta (case 7) at the level of the bifurcation because of small CIA caliber (Figure 4A,B,C, case 7). Endurant II and Valiant stent-grafts (Medtronic Cardiovascular, Santa Rosa, CA, USA) were used for the thoracic and infra-renal abdominal aorta, respectively. Seven conduits were excised at the level of the anastomotic site following the EVAR (Figure 1C, 2D, 2E, 3B). One graft was utilized in situ to make an ilio-femoral bypass for the diseased iliac artery (Figure 4C, case 8). The procedural success rate was 100%. Post-intervention, 3 patients required >2 units of blood transfusion, one of them had a retroperitoneal hematoma (case 5) which was conservatively managed. One patient had local wound infection (case 4), which was successfully treated with a 4-week course of antibiotics. Another patient had a transient ischemic attack on day 2 of the procedure (case 1), which resolved spontaneously. One patient developed thrombotic occlusion of the right limb of the EVAR device on Day-3 of intervention (Figure 2B, 2C) (case 4), which was successfully managed by balloon dilatation and thrombus aspiration (Figure 2D, 2E) via the right transfemoral approach. The median post-intervention hospital stay was 10 days. A repeat CT angiography within 6 months of intervention showed patent access site arteries and also normal intervened aortic segment in all patients. The median follow-up was 18 months. One patient had atherosclerotic total occlusion of the right CIA at 16 months of follow-up (case 2), which was successfully stented (Figure 3C, 3D). He also had atherosclerotic coronary artery disease and left subclavian occlusion. The remaining 7 patients had uneventful long-term follow-up.

**Table 1:** Details of 8 patients treated with retroperitoneal iliac conduit for endovascular aortic repair.

**Case No.**	**Age/Sex**	**Primary disease**	**Comorbidities**	**Dimension of right ilio-femoral segment (mm)**			**Stent-graft size in mm and brand**	**Device introducer sheath size in French (F)**	**Remarks**
				**CFA**	**EIA**	**CIA**			
1	30M	DTA aneurysm	Hypertension	5.3	7	9.5	28 X 24 X 150 mm VALIANT	22	One episode of transient ischemic attack
2	48M	Atherosclerotic DTA aneurysm	Hypertension, smoking CAD, left SCA occluded	7.8	6.9	9.5	36 X 36 X 200 mm VALIANT	24	--
3	74F	Infra-renal AAA	Hypertension, smoking	6.1	5.9	13.8	25 X 16 X 145 mm ENDURANT II	18	Required blood transfusion
4	55F	Infra-renal AAA	Hypertension, smoking	6.4	6.5	8.2	28 X 13 X 145 mm ENDURANT II	18	- Acute thrombosis of right graft limb. Successful balloon angioplasty performed on day 3. -Local site infection treated with extended course of antibiotics.
5	42F	Pseudoaneurysm of DTA	Spinal tuberculosis	6.1	5.2	8.7	28 X 28 X 100 mm VALIANT	22	Retroperitoneal hematoma required blood transfusions
6	39M	Pseudoaneurysm of DTA with impending rupture	Diabetes, hypertension Smoking	6.6	6.4	7.2	30 X 30X 100 mm VALIANT	22	---
7	26F	Pseudoaneurysm of DTA	Pulmonary tuberculosis	5.5	5.0	7.8	22 X 22 X 100 mm VALIANT	22	---
8	52F	Type B aortic dissection	Hypertension	6.42	6.9	7.45	34 X 30 X 150 mm VALIANT	24	Required blood transfusion

**Abbreviations:** AAA: abdominal aortic aneurysm; CAD: coronary artery disease; DTA: descending thoracic aorta; EIA: external iliac artery; CIA: common iliac artery; CFA: common femoral artery; SCA: subclavian artery.

## DISCUSSION

Hostile ilio-femoral anatomy requires an additional retroperitoneal iliac conduit in 2 - 18% of EVAR cases.^4^^-^9 We observed an unfavorable access site in 11% of cases. These include iliac tortuosity, small caliber or diseased arteries, and diffuse severe calcification of access vessels.^5^ Forceful introduction of oversized endovascular devices across compromised ilio-femoral arteries can lead to complications such as arterial rupture, avulsion, hematoma, and retroperitoneal bleeding.^10^ Use of conduits or bypass graft in such hostile vascular access can prevent these and similar devastating complications. Over the years, ROIC has become a standard supplementary procedure in EVAR patients who were previously deferred or subjected to open surgical repair.^3^^-^6 Women have a smaller ilio-femoral arterial system compared to men,^11^ hence they more frequently undergo ROIC compared to men during EVAR.^3^^,^^5^^,^^7^^,^^12^ We had 5 (62.5%) females out of a total of 8 patients subjected to ROIC.

The ROIC is associated with prolonged procedure time, increased need for blood transfusion, local access site complications, and prolonged hospital stay, compared to routine trans-femoral approach.^4^^-^7^,^^12^ We had access site complications such as retroperitoneal hematoma (n=1), local site infection (n=1), and need for blood transfusion (n=3). Certain complications such as acute limb ischemia, fascial dehiscence, anastomotic site leak/rupture, or re-exploration of retroperitoneal area^3^ were not observed in any of our patients. The ROIC is also associated with systemic complications such as pneumonia, renal failure, cardiac arrest, and higher 30-day mortality,^5^ which were not observed in any of our patients. Following completion of the procedure, the conduit is usually snapped at the anastomotic site or otherwise can be used for ilio-femoral bypass graft in those with diseased iliac arteries.^6^ We used it as a bypass graft in one patient following stent-graft deployment, while it was excised in the remaining 7 patients. One patient had subacute limb thrombosis of the device that was unrelated to the residual anastomotic stump.^13^ The post-intervention hospital stay was higher in our cohort compared to others (10 days vs 5-8 days).^5^^,^^12^

Other than ROIC, there are few other alternative techniques to combat difficult vascular access site. Direct aorto-iliac puncture after limited retroperitoneal dissection is one such approach.^14^ Conventional angioplasty and bare-metal stenting of the iliac arteries for a short stenotic segment can be an alternative in selected patients.^15^ For those with diffuse iliac artery narrowing, angioplasty with a 12 mm balloon followed by trans-femoral sheath placement up to a 24 F size can be done for EVAR.^15^ However, this involves the risk of iliac rupture and hematoma formation. Controlled dilatation and stenting of iliac arteries with a covered stent, known as “Endoconduit”, can be another alternative approach.^3^ Following the wide availability of low-profile aortic endografts of 14 - 16 F size (Ovation and INCRAFT),^16^^,^^17^ the limitation of local site access will be further resolved. Until that time, ROIC remains the gold standard and procedure of choice for these patients. Limitations of the present study include a small sample size and retrospective analysis.

In conclusion, we hereby describe our preliminary experience of ROIC in 8 EVAR patients and show favorable short and long-term outcomes. The unique features of our series include 1. Two young tuberculosis patients with thoracic pseudoaneurysm; 2. a pictorial demonstration of cases including those with complications such as acute limb thrombosis of the stent-graft (Figure 2), atherosclerotic disease progression of conduit recipient artery (Figure 3), and use of the conduit for dual purposes (first for device delivery and second as bypass graft to ilio-femoral atherosclerotic disease (Figure 4). We hope these cases will help readers to improve their understanding about use of conduits and their management issues.

## References

[1] Conrad MF, Ergul EA, Patel VI, Paruchuri V, Kwolek CJ, Cambria RP (2010). Management of diseases of the descending thoracic aorta in the endovascular era: a Medicare population study. Ann Surg.

[2] Erbel R, Aboyans V, Boileau C (2014). 2014 ESC Guidelines on the diagnosis and treatment of aortic diseases: Document covering acute and chronic aortic diseases of the thoracic and abdominal aorta of the adult. The Task Force for the Diagnosis and Treatment of Aortic Diseases of the European Society of Cardiology (ESC). Eur Heart J.

[3] van Bogerijen GHW, Williams DM, Eliason JL, Dasika NL, Deeb GM, Patel HJ (2014). Alternative access techniques with thoracic endovascular aortic repair, open iliac conduit versus endoconduit technique. J Vasc Surg.

[4] Abu-Ghaida AM, Clair DG, Greenberg RK, Srivastava S, O’hara PJ, Ouriel K (2002). Broadening the applicability of endovascular aneurysm repair: the use of iliac conduits. J Vasc Surg.

[5] Gupta PK, Sundaram A, Kent KC (2015). Morbidity and mortality after use of iliac conduits for endovascular aortic aneurysm repair. J Vasc Surg.

[6] Lee WA, Berceli SA, Huber TS, Ozaki CK, Flynn TC, Seeger JM (2003). Morbidity with retroperitoneal procedures during endovascular abdominal aortic aneurysm repair. J Vasc Surg.

[7] Etezadi V, Katzen BT, Benenati JF, Alehashemi S, Tsoukas AI, Puente OA (2011). Retroperitoneal versus direct femoral artery approach for thoracic endovascular aortic repair access: a case-control study. Ann Vasc Surg.

[8] Makaroun MS, Dillavou ED, Kee ST (2005). Endovascular treatment of thoracic aortic aneurysms: results of the phase II multicenter trial of the GORE TAG thoracic endoprosthesis. J Vasc Surg.

[9] Matsumura JS, Cambria RP, Dake MD, Moore RD, Svensson LG, Snyder S, TX2 Clinical Trial Investigators (2008). International controlled clinical trial of thoracic endovascular aneurysm repair with the Zenith TX2 endovascular graft: 1-year results. J Vasc Surg.

[10] Vandy FC, Girotti M, Williams DM (2014). Iliofemoral complications associated with thoracic endovascular aortic repair: frequency, risk factors, and early and late outcomes. J Thorac Cardiovasc Surg.

[11] Tran K, Dorsey C, Lee JT, Chandra V (2017). Gender-related differences in iliofemoral arterial anatomy among abdominal aortic aneurysm patients. Ann Vasc Surg.

[12] Tsilimparis N, Dayama A, Perez S, Ricotta JJ (2013). Iliac conduits for endovascular repair of aortic pathologies. Eur J Vasc Endovasc Surg.

[13] Carroccio A, Faries PL, Morrissey NJ (2002). Predicting iliac limb occlusions after bifurcated aortic stent grafting: anatomic and device-related causes. J Vasc Surg.

[14] Parmer SS, Carpenter JP (2006). Techniques for large sheath insertion during endovascular thoracic aortic aneurysm repair. J Vasc Surg.

[15] Peterson BG, Matsumura JS (2009). Tips and tricks for avoiding access problems when using large sheath endografts. J Vasc Surg.

[16] Varkevisser RRB, Swerdlow NJ, Verhagen HJM, Lyden SP, Schermerhorn ML (2020). Similar 5-year outcomes between female and male patients undergoing elective endovascular abdominal aortic aneurysm repair with the Ovation stent graft. J Vasc Surg.

[17] Liang NL, Ohki T, Ouriel K (2021). Five-year results of the INSPIRATION study for the INCRAFT low-profile endovascular aortic stent graft system. J Vasc Surg.

